# Population Exposure to PM_2.5_ in the Urban Area of Beijing

**DOI:** 10.1371/journal.pone.0063486

**Published:** 2013-05-02

**Authors:** An Zhang, Qingwen Qi, Lili Jiang, Fang Zhou, Jinfeng Wang

**Affiliations:** State Key Laboratory of Resources and Environmental Information System, Institute of Geographical Sciences and Natural Resources Research, Chinese Academy of Sciences, Beijing, China; The Ohio State University, United States of America

## Abstract

The air quality in Beijing, especially its PM_2.5_ level, has become of increasing public concern because of its importance and sensitivity related to health risks. A set of monitored PM_2.5_ data from 31 stations, released for the first time by the Beijing Environmental Protection Bureau, covering 37 days during autumn 2012, was processed using spatial interpolation and overlay analysis. Following analyses of these data, a distribution map of cumulative exceedance days of PM_2.5_ and a temporal variation map of PM_2.5_ for Beijing have been drawn. Computational and analytical results show periodic and directional trends of PM_2.5_ spreading and congregating in space, which reveals the regulation of PM_2.5_ overexposure on a discontinuous medium-term scale. With regard to the cumulative effect of PM_2.5_ on the human body, the harm from lower intensity overexposure in the medium term, and higher overexposure in the short term, are both obvious. Therefore, data of population distribution were integrated into the aforementioned PM_2.5_ spatial spectrum map. A spatial statistical analysis revealed the patterns of PM_2.5_ gross exposure and exposure probability of residents in the Beijing urban area. The methods and conclusions of this research reveal relationships between long-term overexposure to PM_2.5_ and people living in high-exposure areas of Beijing, during the autumn of 2012.

## Introduction

Beijing, the capital of China, has experienced a rapid increase in urban population, energy consumption, and vehicle numbers over the past several decades. As once occurred in notoriously foggy London, this development has been followed by more frequent episodes of haze in Beijing [1], [2]. In the composition of the haze, particulate matter less than 10 microns in diameter (PM_10_) is recognized as the main hazard linked to deleterious health effects, such as heart and lung disease [3]. Evidence has shown that when inhaled, fine particles (PM_2.5_) are more toxic and more strongly associated with risks to human health than are coarse particles (between 2.5 and 10 microns in diameter) [4], [5]. Therefore, overexposure to PM_2.5_ poses a nontrivial risk to public health in Beijing.

A large number of case studies have associated the short-term exposure of limited individuals to PM_2.5_ with aggravation of asthma, pulmonary dysfunction, lung cancer, heart disease, stroke, and other illnesses [6]–[8]. Some studies have suggested that long-term continuous PM_2.5_ exposure is significantly related to deteriorating health conditions and increased morbidity [9]–[11]. Other studies have shown the accumulated health effects of PM_2.5_ and have analyzed annual statistical data of long-term continuous PM_2.5_ pollution, together with its impact on mortality, morbidity, and life expectancy. These studies are usually based on case studies and ignore temporal and spatial differences [12], [13]. With regard to the health of a population, the importance of medium-scale and discontinuous exposure to PM_2.5_ should be recognized, because this is connected to cumulative temporal effects and time variations associated with meteorological conditions. This approach is more likely to ascertain the spatiotemporal regularity in the relationship between public exposure and response, which is highly relevant to every person subject to urban particulate pollution.

To determine the spatiotemporal regularity and to monitor environmental health, high-resolution temporal and spatial measurement of atmospheric pollutants is of crucial importance [14]. Some investigators have monitored several sampling sites in Beijing in an attempt to analyze the spatial distribution and time-varying characteristics of PM_2.5_ [15], [16]. However, these analyses were based on discrete points and low time-resolution sampling, which affects the statistical average values. Furthermore, they did not address the population adequately; hence, exposures were not studied [15], [16]. To investigate the risk from PM_2.5_ for the total population of Beijing, a spatially continuous study with high temporal resolution sampling is necessary to detect hotspots, estimate vulnerability, and accurately assess population exposure.

In October 2012, the Beijing Environmental Protection Bureau (BJEPB) released hourly monitoring data from 35 sampling sites, covering a large area of the city. In this study, we collected data from 37 of those days. We also acquired and manipulated the latest census data of population distribution, based on appropriate population units. We performed a 37-day interpolation over the entire city, using the daily averages at these sites. We constructed time series charts of daily air quality index (AQI) levels for the entire city, and related them to PM_2.5_ concentration. To analyze population exposure to PM_2.5_ in the Beijing urban area, especially in autumn, we used spatiotemporal statistical methods and made a distribution map of accumulated exceedance days, population exposure, and exposure probability exceeding the AQI standards.

## Methods and Data

### Source of PM_2.5_ data

**Figure 1:**
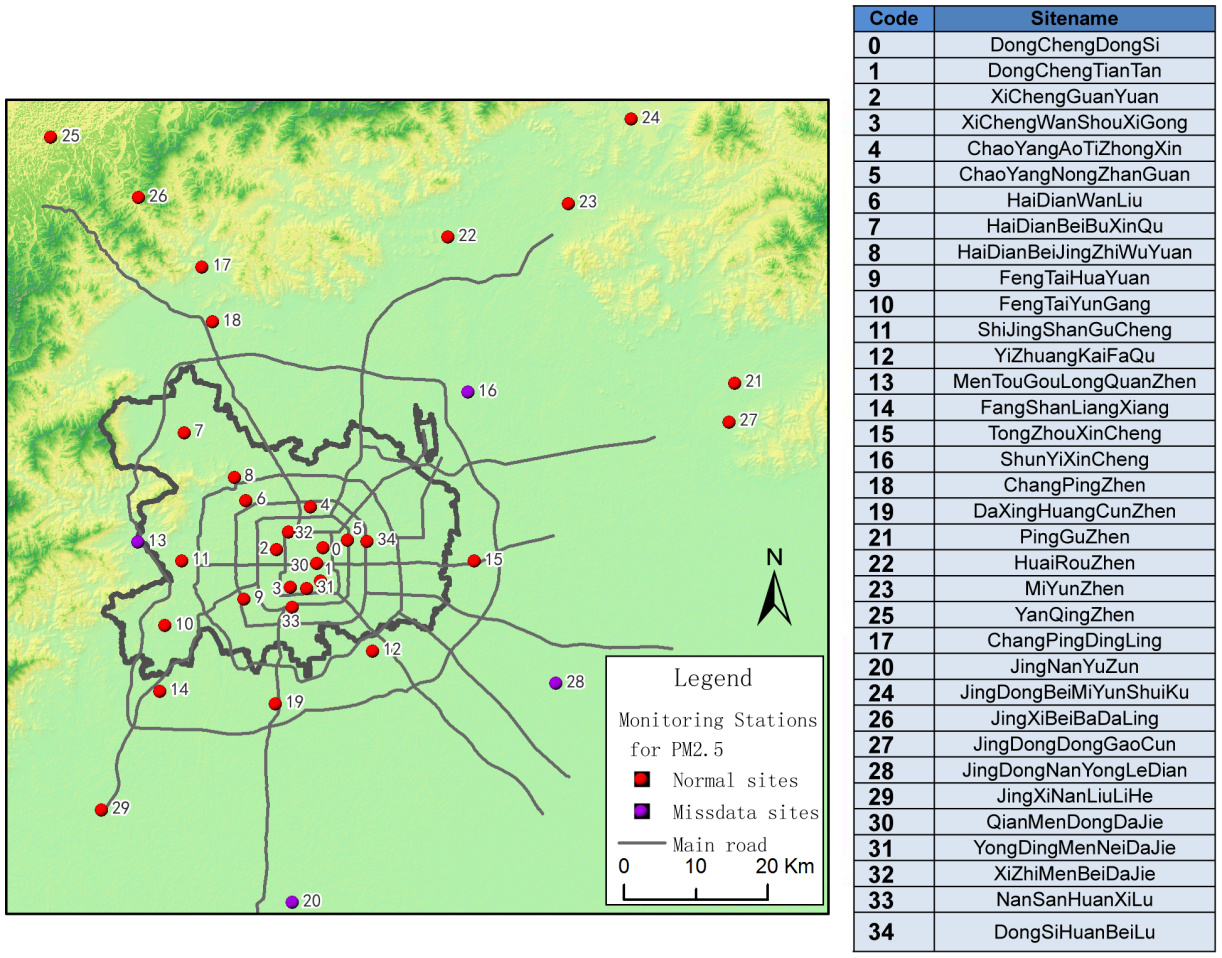
Locations of monitoring stations for PM_2.5_ in Beijing from BJEPB.

To strengthen ambient air pollution monitoring, 35 automatic monitoring stations for PM_2.5_ fine particles were established in Beijing by BJEPB. At the end of September 2012, BJEPB gradually released these data in real time to the public. Figure 1 shows the locations of the BJEPB monitoring stations.

Each monitoring station automatically acquires hourly PM_2.5_ concentration data. We collected the 37-day dataset from October 8 to November 13, 2012, for use as experimental data, from the Centre of the City Environmental Protection Monitoring Website Platform (www.bjmemc.com.cn), which is maintained by BJEPB. Daily 24-hour average concentration data were calculated based on the hourly real-time data. Thus, we obtained daily average PM_2.5_ concentrations, between October 8 and November 13, 2012, for 35 sites in Beijing (see Table S1). The unit of concentration is µg/m^3^ (micrograms per cubic meter). In addition, data on wind direction and scale for Beijing were also acquired for the same period from the Beijing Meteorological Bureau (see Table S2). Owing to equipment failure and transmission problems, some site data were incomplete or lost; thus, ultimately, we selected 31 of the 35 sites that had a complete record as our data source. The four omitted data sites are: Nos.13, 16, 20, and 28 (see Figure 1).

### Census data

Beijing is the capital of the People's Republic of China, and has an area of 16,410 square kilometers. The latest Sixth National Population Census of the People's Republic of China [17], also known as the 2010 Chinese Census, was used in our analysis. This census revealed an official population for Beijing of 19,612,368. Its urban area includes six districts (county level): Xicheng, Dongcheng, Haidian, Chaoyang, Shijinshan, and Fengtai. The total urban area comprises 1378 square kilometers with a 2010 census population of 11,683,213.

**Figure 2:**
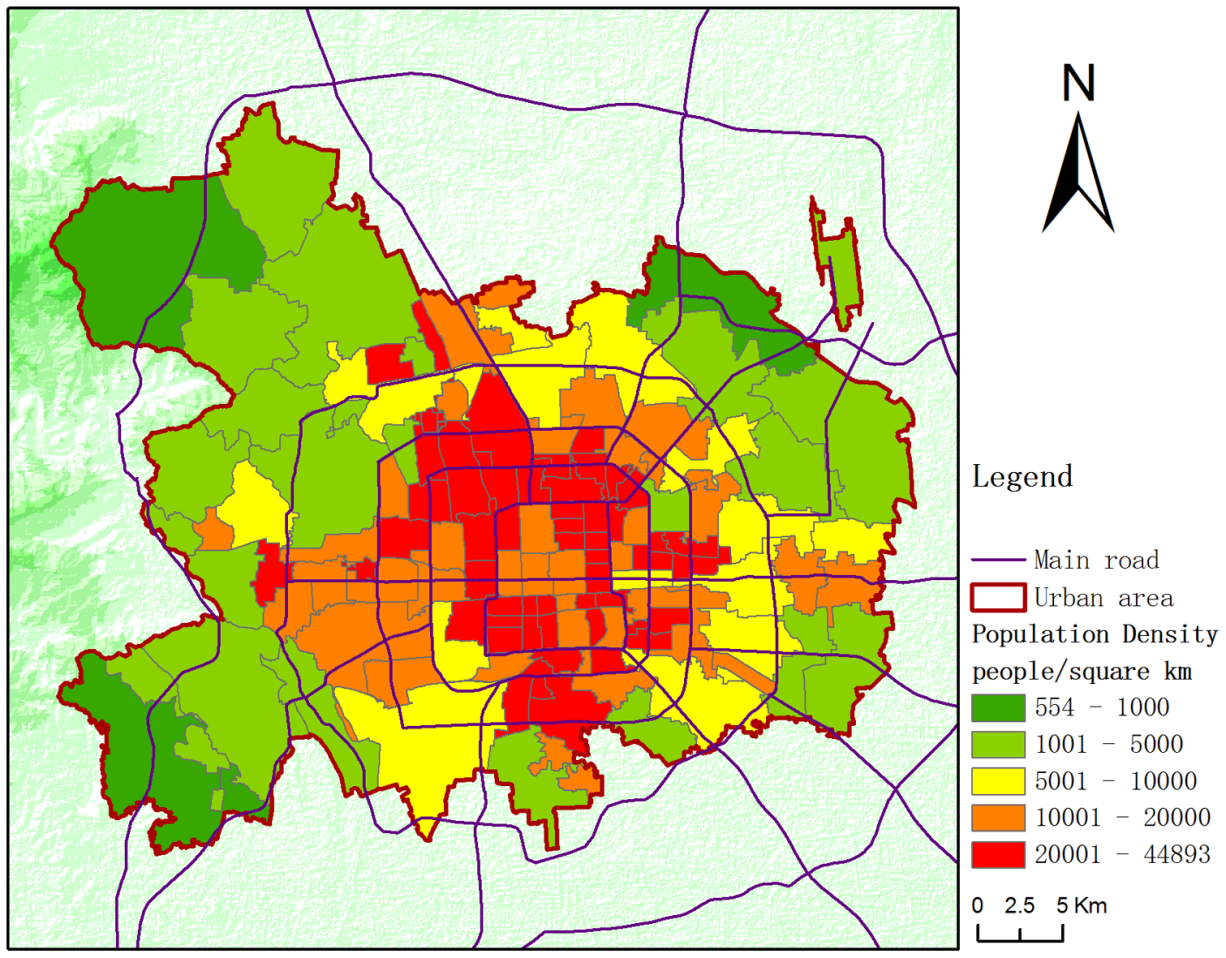
Census geographic unit population densities of Beijing urban area.

To improve analysis precision, towns and sub-districts (street level) were chosen as the appropriate census geographic units. Using the Beijing Administrative Districts Atlas and Tabulation on the 2010 Population Census of Beijing Municipality (town and sub-district volume) [18], [19], we matched the census data of the Beijing urban area to the administrative map. After data processing and merging, 129 census geographic units (street level) were formulated with the census data and area (see Table S3). Figure 2 shows the population density of these geographic units.

### Spatial interpolation for PM_2.5_ concentrations

**Figure 3:**
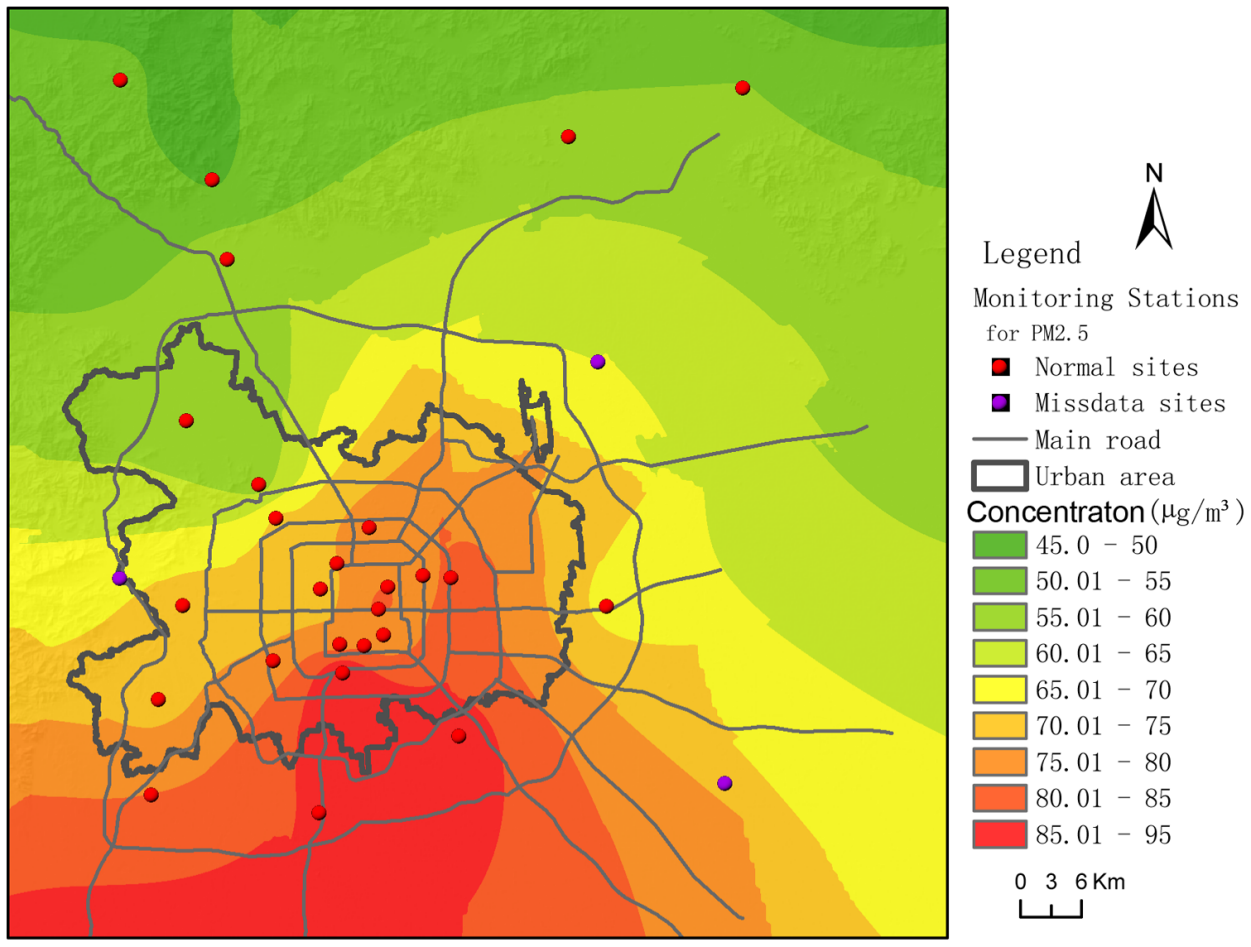
Sample of spatial interpolation for PM_2.5_ concentrations (October 9, 2012).

The original PM_2.5_ concentration data were at discrete points, i.e., at the automatic monitoring stations. We needed to interpolate the site data into surface data to characterize concentrations over the entire area. There are numerous spatial interpolation methods. Kriging, originated by Krige in 1951 and developed by Matheron [20], is one popular method used in various fields [21]. Kriging can interpolate the value of a random field at an unobserved location from observations of its value at nearby locations. Kriging has various forms, including: ordinary Kriging (OK), simple Kriging, indicator Kriging, universal Kriging, and intrinsic Kriging [22]. Of these, OK is the one used most frequently. It assumes a constant but unknown mean, which allows construction of an unbiased estimator that does not require prior knowledge of the stationary mean of the observed values [22]. Here, we use an OK method to interpolate spatially the PM_2.5_ concentrations. Figure 3 shows a sample of one such spatial interpolation on October 9, 2012. The same method and parameters of interpolation were applied to all 37 days of daily site concentration data; ultimately, obtaining 37 daily averaged, areally interpolated concentrations.

### Cumulative exposure hotspot detection for PM_2.5_ air pollution

A series of epidemiology studies reported robust associations between short-term [6]–[8] and long-term exposure to PM_2.5_ and adverse health effects, including cardiac and respiratory morbidity and mortality [23]. The focus of our study is on long-term cumulative effects. However, the estimation of the level of direct exposure of the population cannot be made from single-day concentrations. Harm from PM_2.5_ concentration would not usually be immediately reflected, and there would be certain hysteresis and cumulative effects. Moreover, annual average concentration or periods of average concentration cannot indicate the risk level from PM_2.5_. This is because average concentration does not reflect the difference between a normal concentration and its exceedance concentration. Furthermore, it is difficult to use average concentration to reveal spatiotemporal differences.

**Figure 5:**
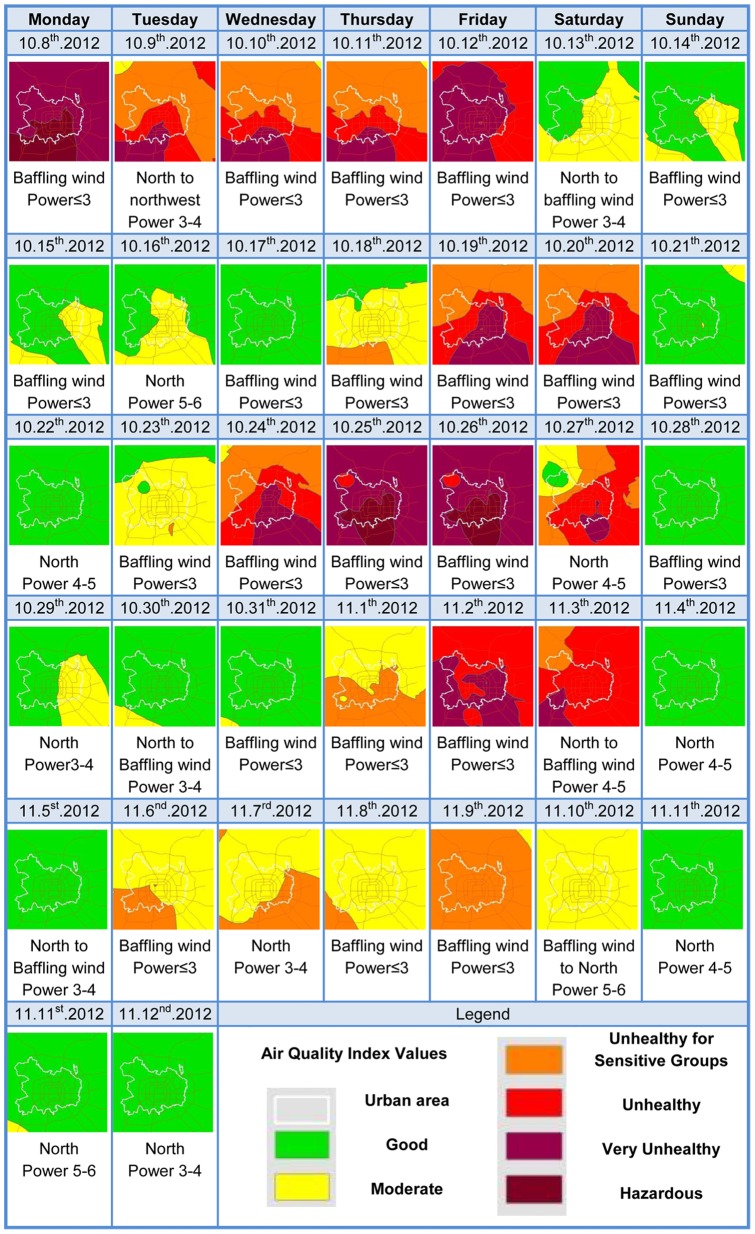
Thirty-seven daily Air Quality Index change in Beijing urban area.

In addition, during the observation period, PM_2.5_ pollution in Beijing showed a certain periodicity. By arrangement of the AQI-level diagrams in calendar order (Figure 5), each week appeared to have an independent and complete process of pollution. Except for October 8 (the first working day after the Chinese National Day holiday), the most serious pollution within the week always occurred on a Friday.

Therefore, we adopted a method for the evaluation of the cumulative effect. To calculate the level of exposure of human beings to particulate matter concentration, we must determine air pollution levels. The AQI helps in understanding the impact of local air quality on health. It is calculated from concentrations of the primary pollutant, which for Beijing in autumn is usually PM_2.5_. According to the standard, and for ease of understanding, individual AQIs of PM_2.5_ were divided into six categories by considering different concentration ranges (Table 1). Each category corresponds to a different level of health concern. We can convert the concentration of PM_2.5_ to AQI levels with the help of the Ambient Air Quality Standards (GB 3095–2012) and Technical Regulation on Ambient Air Quality Index (HJ 633–2012, on trial) in Table 1.

**Table 1:** Individual Air Quality Index standards of PM_2.5_ *.

Individual Air Quality Index (AQI) Values	AQI Levels	Levels of Health Concern	24 Hour Average PM2.5 Concentrations Range (µg/m^3^)	Health Implications
0 to 50	Level 1	Good	0 to35	Air quality is considered satisfactory, and air pollution poses little or no risk.
51 to 100	Level 2	Moderate	35 to 75	Air quality is acceptable, but for some pollutants, there may be a moderate health concern for a very small number of people. People who are unusually sensitive to ozone may experience respiratory symptoms.
101 to 150	Level 3	Unhealthy for Sensitive Groups	75 to 115	Although the general public is unlikely to be affected in this AQI range, people with lung disease, older adults and children are at a greater risk from exposure to ozone; those with heart and lung disease, older adults and children are at greater risk from airborne particles.
151 to 200	Level 4	Unhealthy	115 to 150	Everyone may begin to experience adverse health effects, and members of the aforesaid sensitive groups may experience more serious effects.
201 to 300	Level 5	Very Unhealthy	150 to 250	This situation would trigger a health alert, signifying that everyone may experience more serious health effects.
301 to 500	Level 6	Hazardous	above 250	This would trigger a health warning of emergency conditions, and the entire population is more likely to be affected.

* From Ambient Air Quality Standards (GB 3095–2012) and Technical Regulation on Ambient Air Quality Index (HJ 633–2012, on trial) from the website of the Ministry of Environmental Protection, China (http://kjs.mep.gov.cn/hjbhbz/bzwb/dqhjbh/dqhjzlbz/201203/W020120410330232398521.pdf and http://kjs.mep.gov.cn/hjbhbz/bzwb/dqhjbh/jcgfffbz/201203/W020120410332725219541.pdf).

We transformed the 37 daily averaged, areally interpolated concentrations to maps of AQI- level polygons, obtaining 37 AQI-level map layers. Through overlaying these map layers, an intersection layer was generated. Polygons in the intersection layer are minimum exposure units. An AQI of PM_2.5_ above the value of 100 (above level two) is considered as the urban air quality standard according to Ambient Air Quality Standards (GB 3095–2012), for which the 24-hour average PM_2.5_ concentration exceeds 75 µg/m^3^ [24], [25]. Equation 1 is used to calculate the cumulative days exceeding the reference concentration of China's air quality guidelines in each minimum exposure unit:
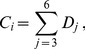
(1)where 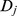 denotes days at 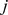 air quality level in 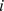 minimum exposure units. 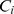 refers to the cumulative days under AQI levels from 3 to 6 exposure (PM_2.5_ concentration exceeding 75 µg/m^3^).

After calculation, the number of cumulative exceedance days in each minimum exposure unit is obtained. This facilitates the determination of areas of continued high exposure.

### Area-weighting method estimating population exposure to PM_2.5_

To estimate the level of exposure of the population to PM_2.5_, we should ascertain which areas are exposed to high concentrations of PM_2.5_ and the size of their populations. If there is no risk, then there is no exposure, and if there is no population, there is no exposure [9].

Based on a hotspot analysis, we can develop statistics regarding populations under continuous exposure. Previous studies used census geographic units as the smallest population exposure unit, but such a unit often has a different risk exposure level. To solve this problem, the area-weighting method was used to calculate the population under exposure. This method involves a straightforward algorithm for areal interpolation. Based on the assumption that population is evenly distributed within a source zone, the constant population density of each zone is initially estimated. Then, the size of each overlapping area between target and source zones is used as a weight to estimate the population of the target zones [26].

To obtain cumulative population exposures exceeding the AQI standards, we assume that the population in urban internal areas is evenly distributed among the internal streets. The population density of every census geographic unit (street) was calculated separately using Equation 2:
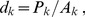
(2)where 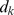 denotes census geographic unit 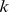 population density, 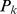 census geographic unit 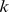 population, and 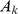 census geographic unit 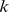 area.

Population exposures for each level in every census geographic unit are calculated using Equation 3:
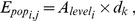
(3)where 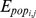 indicates census geographic unit 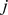 under AQI level 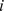 population exposure, 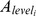 is the area under AQI level 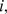 and 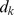
is census geographic unit 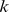 population density.

Then, the total population exposures under AQI level 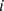 are calculated with Equation 4.
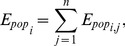
(4)where 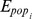 designates the total population exposures under AQI level 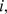 and n is the number of census geographic units.

Total population exposures exceeding the AQI standard are calculated using Equation 5.
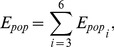
(5)where 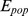 represents the population exposures exceeding the AQI.

Then, the percentage of population exposures of the total population under AQI level 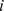 is calculated using Equation 6.
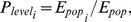
(6)where 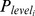 denotes the percentage of the population exposures of the total population under AQI level 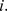


**Figure 4:**
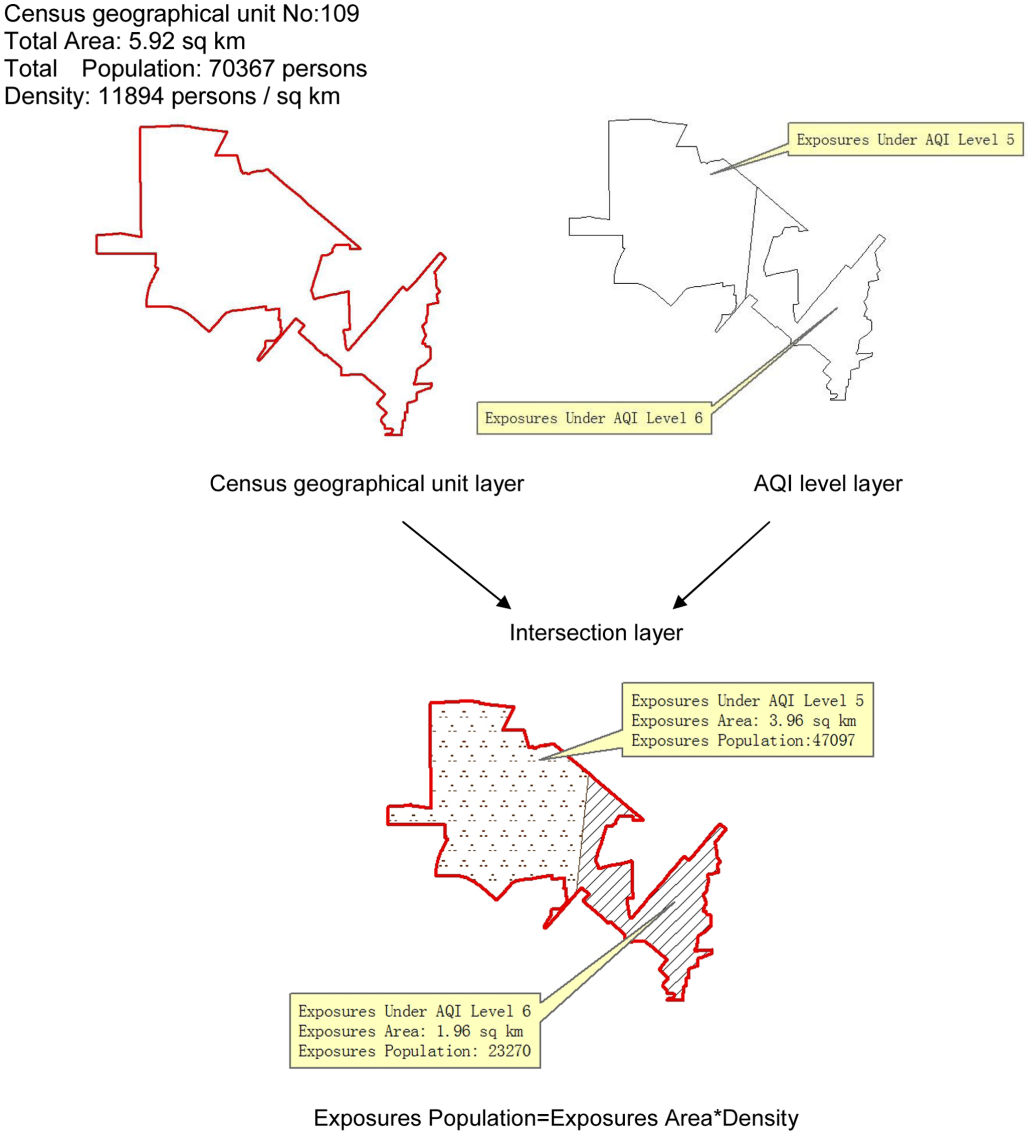
Population exposures estimation method.

Figure 4 illustrates the estimated population exposure method for one census geographical unit.

## Results

Figure 5 shows that during the 37 days, PM_2.5_ pollution in Beijing had some spatiotemporal variations. The pollution process showed that southern Beijing began to accumulate PM_2.5_ and exceed the standard, which was then followed gradually by northern Beijing. Following a change of wind direction, wind scale, humidity, other weather variables or related conditions, particulate pollution declined from north to south with the northern part of the city being the first to meet the levels of the standards. The third week of the time series charts depicts a complete, typical and long-lasting pollution process.

**Figure 6:**
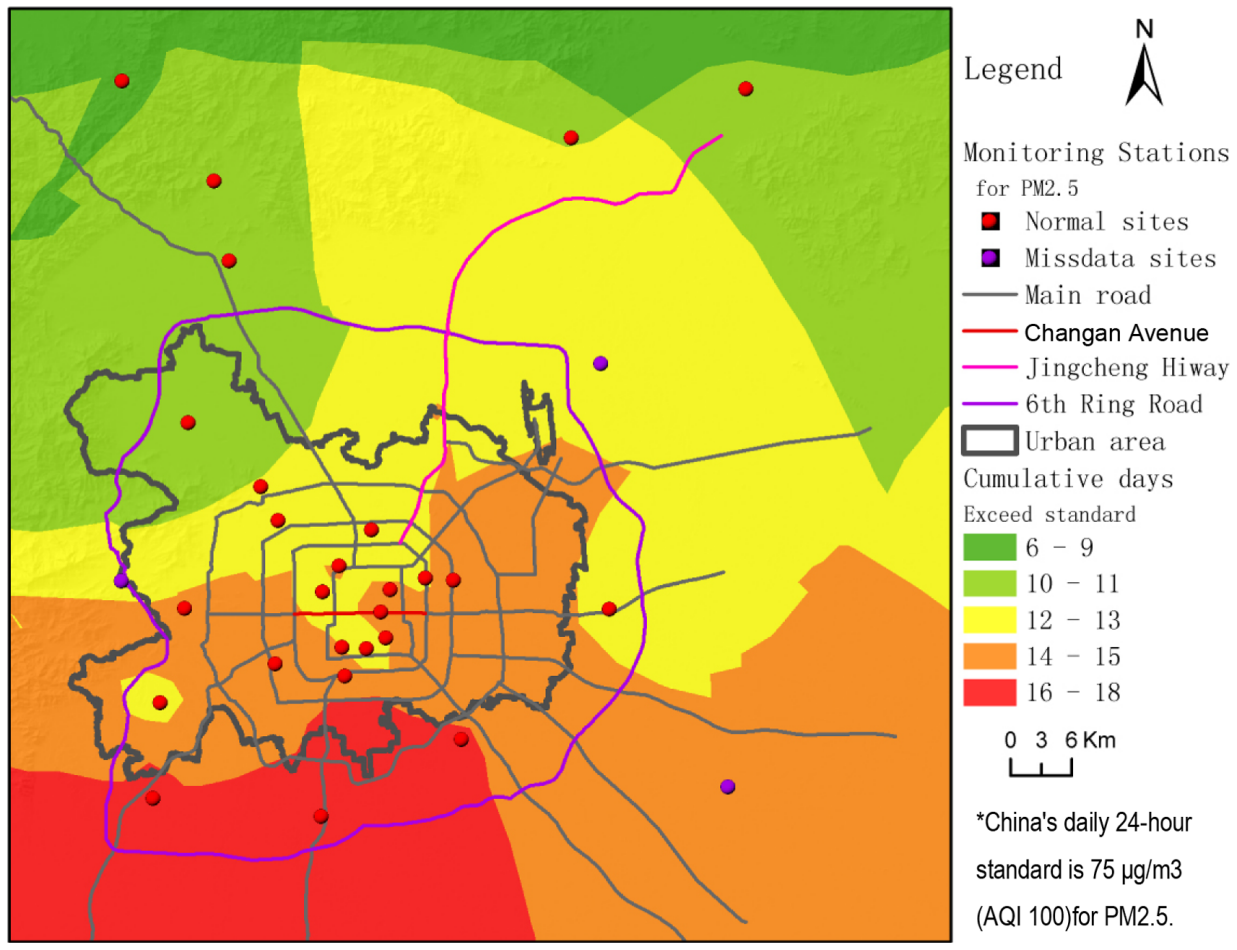
Number of cumulative exceedance days.

In our second result (Figure 6), it is not difficult to determine that Beijing suffers severe pollution of PM_2.5_. The citywide cumulative number of exceedance days is generally high. Throughout the 37-day monitoring period, the number of exceedance days inside the 6th ring road was above ten. Meanwhile, the statistical result in this figure also displays an obvious spatial differentiation; the south and the southeast regions are hotspots of PM_2.5_ pollution. Areas south of Chang An Avenue and east of the Beijing-Chengde expressway seem to be the high risk areas with up to two weeks or more of exceedance days. A large area south of the 4th ring road accumulated 16–18 days during this 37-day monitoring period. In other words, for half of the time or more, the people living there were exposed to excessive PM_2.5_ pollution.

**Figure 7:**
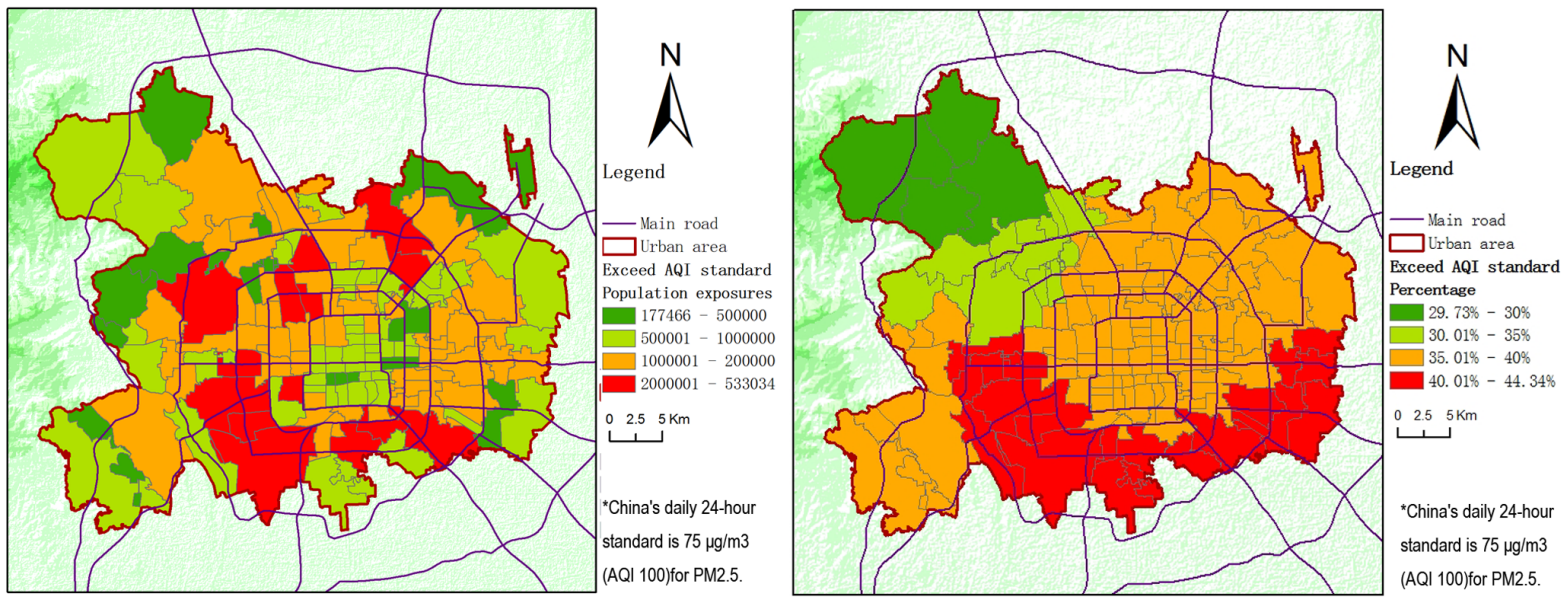
Population exposures (left) and percentages (right) exceeding AQI standard.

Statistically, over the duration of data collection, the cumulative exposed population is 160,561,842 (average daily exposed population is 4,339,509), representing 37.14% of Beijing's cumulative total urban population. The cumulative population exposed to hazardous levels of PM_2.5_ (AQI level 6) is 12,315,860 (average daily exposed population is 332,861), or 2.85% of the total (see Table S3). As shown in Figure 7, the urban area between the second and fifth ring roads had generally high exposure. In contrast with the accumulated amount of exceedance days, the hotspots of accumulative exposed population were not just the areas to the south and southeast of Beijing; north to Chang An Avenue and west to the Beijing-Chengde expressway, there were also some census units in such hotspots of accumulated exposure.

## Discussion

The spatiotemporal variations of PM_2.5_ pollution in Beijing might be related to topography. West of Beijing is Xishan Mountain, part of the Taihang Mountains. North of the city is Jundu Mountain, part of the Yanshan Mountains. Surrounding the Beijing plain, these two mountains form a semicircular arc, extending to the southeast. The dominant wind direction during our monitoring period was northerly and northwesterly. Daily emissions from Beijing disperse towards the opening of the mountain arc, over the flat plain. When the wind loses strength or a weak southerly wind develops, atmospheric stability increases rapidly. Then, fine particulates accumulate from the south to north, as shown by the time series. Moreover, the provinces south of Beijing, such as Hebei, Henan and Shandong, receive large quantities of emissions every day. With wind transport from remote areas and increasing local background values, the pollutant accumulation and north-south spatial differences described above, become increasingly likely. Later in the pollution process, there is often a northerly wind of force 3 and above sweeping across the entire city, and with strong pollutant dispersal over the flat northeastern area, a pattern of reduction in levels of pollution appears from north to south.

The periodicity of PM_2.5_ pollution in Beijing is probably linked to variations of meteorological conditions, as well as to periodic changes of transportation emissions caused by travel regulations in the city. This remains to be verified by our follow-up studies. Beijing is still expanding rapidly with tremendous daily emissions by traffic and other local sources. Combined with emissions from nearby provinces, it is easy for PM_2.5_ pollution to exceed standards. Such conditions result in the high number of accumulated exceedance days. The spatial differences shown in Figure 6 might be explained by the spatiotemporal regularity of pollution processes. That is, the south is always the first area to exceed normal concentrations and the last to revert below them, causing a longer duration of pollution in that area compared with the north.

The northern part of the city saw fewer accumulative numbers of days of exceedance than the southern part. Nevertheless, given the spatial distribution of total population exposure and the dense population in northern areas, its 37-day cumulative exposure still easily reached the highest level. That is, the north may not have less population and economic harm from PM_2.5_ pollution than the south. If the impact of population density is not considered, the exposure shows that the south still has a greater accumulated probability of exceedance days than the north.

Because we only collected PM_2.5_ data for 37 days in autumn from the Beijing Environmental Protection Bureau, the above analysis and results may not be applicable to other seasons, because air pollution emissions and meteorological conditions change significantly between seasons in Beijing. We need to gather PM_2.5_ data over longer periods to perform a long time series analysis.

## Conclusions

Through spatiotemporal analysis of PM_2.5_ data for 37 days in autumn, and by overlaying the population distribution of the Beijing urban area, we discovered hotspots in which cumulative days exceeded the AQI standard, and we calculated the population exposures exceeding that standard. The concentration levels of PM_2.5_ have become a serious problem in Beijing that requires attention.

The research methods and conclusions here reveal relationships between long-term overexposure to PM_2.5_ and people living in high-exposure areas during the autumn in Beijing. The research results will also support future studies, especially into the adverse health effects of long- and short-term exposure to PM_2.5_.

## Supporting Information

Table S1 **35 sites daily average PM_2.5_ concentration from October 8, 2012 to November 13, 2012 in Beijing.**
(XLS)

Table S2 **Wind direction and Scale from October 8, 2012 to November 13, 2012 in Beijing.**
(XLS)

Table S3 **Census geographic units population and exposed population.**
(XLS)
